# Editorial: New developments in understanding brain and cerebromicrovascular aging: Toward prevention of vascular cognitive impairment and Alzheimer's disease

**DOI:** 10.3389/fnagi.2022.1020271

**Published:** 2022-09-16

**Authors:** Madison Sanford, Sharon Negri, Stefano Tarantini

**Affiliations:** ^1^Department of Biochemistry and Molecular Biology, Center for Geroscience and Healthy Brain Aging, University of Oklahoma Health Sciences Center, Oklahoma City, OK, United States; ^2^Peggy and Charles Stephenson Cancer Center, University of Oklahoma Health Sciences Center, Oklahoma City, OK, United States; ^3^Department of Biology and Biotechnology “Lazzaro Spallanzani”, Laboratory of General Physiology, University of Pavia, Pavia, Italy; ^4^Hudson College of Public Health, University of Oklahoma Health Sciences Center, Oklahoma City, OK, United States

**Keywords:** neurovascular, brain aging, VCID, ADRD, Alzheimer's disease

Cardiovascular and cerebrovascular diseases are the most common cause of death among older people in the United States, accounting for ~1/3 of all deaths in the US at the age of 65, and nearly 2/3 at the age of 85. With a projected increase in the number of adults over 65 years old from 12 to 22% in the next 30 years, addressing age-related vascular diseases is of critical importance, as the annual cost to care for the older population is expected to more than double in that same time frame. Aging in the brain is characterized by a vast array of functional and structural alterations of the microcirculation, contributing to the pathogenesis of a range of age-related diseases including vascular cognitive impairment (VCI), Alzheimer's disease (AD), and mild cognitive impairment (MCI). The collection of articles published in the Research Topic titled: “*New Developments in Understanding Brain and Cerebromicrovascular Aging: Toward Prevention of Vascular Cognitive Impairment and Alzheimer's Disease*” highlights the recent growing interest and understanding of the role of the aging vasculature in the context of the age-related loss of cognitive function. The goal of this collection is to stimulate interest and gather evidence that relates to the mechanisms underlying the neurodegenerative diseases that associate with aging, with particular emphasis on treatment and interventions that aim to prevent or delay the onset of vascular cognitive impairment and Alzheimer's disease.

VCI and AD are the most common forms of cognitive disorder associated with cerebrovascular diseases and are related to increased morbidity and mortality among the older population (Wiesmann et al., 2013). Growing evidence highlighted in this Research Topic emphasizes the multifactorial nature of age-related cerebrovascular disease. Despite the evidence that reversal of vascular dementias has shown mixed results, we now understand that many of the risk factors are largely preventable. Such risk factors examined in this special issue include the effects of obesity, sedentary lifestyle, eating behaviors, hypertension, diabetes, circulating endocrine factors, and others. The current challenges and efforts in the field will also be briefly explored, while novel biomarkers of cerebrovascular disease and AD will be discussed.

Bliss et al. investigate the effects of aerobic exercise training on cerebrovascular and cognitive function in sedentary, obese, older adults. In their study, the authors showed that cerebrovascular function and cognition improved following 16 weeks of exercise and determined the presence of a dose-response relationship between the amount of exercise sessions performed and cerebrovascular reactivity to cognitive stimuli. Lifestyle interventions aimed at delaying or preventing age-related pathophysiology include exercise (Lucas et al., 2015; Bliss et al., 2021) and alterations in diet (Dobreva et al., 2022; Maroto-Rodriguez et al., 2022) [i.e. caloric restriction, intermittent fasting (Balasubramanian et al., 2020; Bray et al., 2022), or methionine restriction]. These highly translatable interventions have been shown to be effective in mediating increased health and lifespan in mice and other model organisms. Thelen and Brown-Borg reviewed the existing evidence to better understand the therapeutic potential of diets to act as a future treatment option for AD patients.

Circulating insulin-like growth factor-1 (IGF-1) deficiency is a well-known predictor of cognitive decline. For instance, previous studies established a causal link among age-related decline in circulating levels of IGF-1, neurovascular dysfunction, and cognitive impairment (Tarantini et al., 2021). Miller et al. further describe how IGF-1 deficiency is associated with increased susceptibility to cerebral microhemorrhages and signs of microvascular degeneration in the retina in response to hypertension. The formation of microhemorrhages, which associate with cognitive impairments, psychiatric disorders, and gait dysfunctions in patients is also caused by mild traumatic brain injury (mTBI). In this context, Toth et al. have investigated the effect of mTBI on cerebral microhemorrhages in aging and have reported that aging enhances the formation of parietal and occipital microhemorrhages after mTBIs.

The properties of the brain cerebral microvasculature can be also studied in the retina (Newman, 2013), as those micro vessels are closely related to those in the brain (Ptito et al., 2021). In addition to the work from Miller et al., illustrated above, Cheng et al. utilized the retina as model to study the association between diabetic retinopathy and cognitive impairment in this extensive systematic review. This timely work better examines the correlation of diabetic retinopathy with cognitive impairment, which has not been well-studied yet.

Normal brain function is dependent on moment-to-moment adjustment of cerebral blood flow to match the increased demands of active brain regions (Masamoto and Vazquez, 2018; Yabluchanskiy et al., 2021). The underlying biological mechanism termed neurovascular coupling (NVC) is dependent on the production of the endothelium-derived vasodilator molecule nitric oxide in response to multiple mediators released from activated astrocytes. Csipo et al. studied how geriatric sepsis affects endothelial dysfunction and impaired NVC responses precede cognitive impairment in a mouse model of geriatric sepsis, suggesting that sepsis-associated endothelial dysfunction and impairment of NVC responses may contribute to long-term cognitive deficits in older sepsis survivors. In addition, Liu et al. aimed to explore the characteristics and contributions of cerebral hemodynamics and carotid atherosclerosis to cognitive dysfunction. The authors discovered that pathological changes in macrovascular structure and function are correlated with cognitive impairment in dementia. This is intriguing as recent studies have shown that macrovascular aberrations closely associated with decreased microvascular health in aging (Xu et al., 2017). Shabaan et al. were also interested in examining the link between cognition and cerebrovascular reactivity in midlife women with both preeclampsia and maternal vascular malperfusion (MVM). Their data suggested that MVM in women with preeclampsia is a promising sex-specific indicator of cerebrovascular integrity in midlife (Shabaan et al.).

Another challenge in the field of cerebromicrovascular aging has been identifying a reliable indicator or biomarker to detect patients with different forms of dementia at an early stage (Zampino et al., 2022). Liu et al. showed that basal ganglia perivascular spaces were associated with increased cardiovascular risk burden and regional differences in cerebral blood flow and gray matter volume, thereby advancing the idea that perivascular spaces are an important associated phenotypic indicator of VCI with a larger population of cognitively intact individuals. Similarly, Yao et al. suggested that in a population of frail patients with cardiovascular disease the urinary 8-oxoGsn (a typical marker of oxidative modification of RNA) adjusted by urinary creatine levels, may be a useful indicator for the early screening of MCI. In their study, Sun et al. searched for an endothelial-specific biological marker to better understand the pathogenesis of cerebral small vessel disease (CSVD). Intriguingly, they found a relationship between ADAMTS13 activity and white matter hyperintensity (WMH), subcortical infarction, but not with cerebral microhemorrhage. In addition, ADAMTS13 (which regulates the activity of endothelium-derived von Willebrand factor by cutting it into smaller, less active molecules) may play an essential role in the progression of CSVD. Another typical marker that associates with CSVD are WMHs. In their study Bauer et al. perform an extensive assessment of WMH volume and location. Notably, their results suggest that white matter microstructure may be a better predictor of WMHs volume than either brain iron levels or cerebral blood flow (CBF) but also draws attention to the possibility that some early WMH markers may be location specific. Furthermore, Vettore et al. add that the association between WMH burden and connectivity strength, during resting-state functional networks, is different between amnestic and non-amnestic MCI patients. Despite the exploratory nature of this study, these results suggest that clinical profiles reveal mechanistic interactions that may play a critical role in the classification of diagnostic vs. prognostic conditions. In another study, Liu et al. developed an innovative transfer learning model based on speech and natural language processing (NLP) technology to effectively improve the early diagnosis of AD.

Genetic variability is another interesting factor that can play a major effect in the development of cerebral vasculature (Bogorad et al., 2019), and as a determinant for age-related cerebrovascular disease and AD, as demonstrated in both clinical and experimental studies (Korstanje et al., 2021; Kulminski et al., 2022). Recent genetic mutation studies done in rodents have elucidated how different genetic backgrounds can significantly affect flow-mediated outward remodeling in the bilateral posterior communicating arteries after unilateral occlusion of a middle cerebral artery. Therefore, Eto et al. aimed to investigate the relationship between anatomical variations in the circle of Willis in cerebrovascular disease. This study sets up an important framework in which future investigations can further expand to understand what genetic variants are critical in determining anatomical variations in the circle of Willis, thus increasing vulnerability to age-related vascular disease. Additionally, Ehret et al. draw attention to an important point mutation in *Notch3* (N3), which is known to cause Cerebral Autosomal Dominant Arteriopathy with Subcortical Infarcts and Leukoencephalopathy (CADASIL). N3 is expressed in neural stem and progenitor cells in the hippocampus, where the authors previously demonstrated that it is a critical regulator of precursor cell proliferation and differentiation in the neurogenic niche of the murine hippocampus. Based on the previous results, they now suggest that N3 might exert regulatory influences on neuronal plasticity that could impact hippocampus-dependent learning and memory. The hippocampus together with the pre-frontal cortex constitute a very important brain area involved in the regulation of emotion and cognition. Qi et al. described how a bilateral hippocampal microinjection of streptozotocin can induce AD-like behavioral performance in mice, and adaptive changes in synaptic plasticity against neuroinflammatory and endocrinal injuries. The authors interpret these findings and hypothesize the underlying mechanisms to be associated with the inadequate balance in the hippocampal expression of the key proteins involved in Wnt signaling pathway.

Lastly, Takeuchi et al. discovered that the RNA transcription analysis of metabolism related genes in circulating white blood cells (WBCs) has the potential to provide significant information relating to impaired cell-cell interaction between WBCs and endothelial cells of aged mice. Additionally, this can serve as a tool to evaluate the change of the cell-cell interaction caused by various treatments or diseases.

## Author contributions

ST: Conceptualization, funding acquisition, writing-original draft, and writing-review and editing. MS and SN: Writing-review and editing. All authors contributed to the article and approved the submitted version.

## Funding

This work was supported by grants from the National Institute on Aging (NIA R03AG070479 and NIA K01AG073614), the American Heart Association AHA CDA941290, the NIA-supported Geroscience Training Program in Oklahoma (T32AG052363), the NIA-supported Oklahoma Nathan Shock Center, and the NIGMS supported Center of Biomedical Research Excellence (CoBRE) (1P20GM125528-01A1).

## Conflict of interest

The authors declare that the research was conducted in the absence of any commercial or financial relationships that could be construed as a potential conflict of interest.

## Publisher's note

All claims expressed in this article are solely those of the authors and do not necessarily represent those of their affiliated organizations, or those of the publisher, the editors and the reviewers. Any product that may be evaluated in this article, or claim that may be made by its manufacturer, is not guaranteed or endorsed by the publisher.
